# Tissue-type plasminogen activator selectively inhibits multiple toll-like receptors in CSF-1-differentiated macrophages

**DOI:** 10.1371/journal.pone.0224738

**Published:** 2019-11-07

**Authors:** Lipsa Das, Pardis Azmoon, Michael A. Banki, Elisabetta Mantuano, Steven L. Gonias

**Affiliations:** Department of Pathology, University of California San Diego, La Jolla, California, United States of America; University of Toledo Health Science Campus, UNITED STATES

## Abstract

Tissue-type plasminogen activator (tPA) is a major activator of fibrinolysis, which also attenuates the pro-inflammatory activity of lipopolysaccharide (LPS) in bone marrow-derived macrophages (BMDMs) and *in vivo* in mice. The activity of tPA as an LPS response modifier is independent of its proteinase activity and instead, dependent on the N-methyl-D-aspartate Receptor (NMDA-R), which is expressed by BMDMs. The major Toll-like receptor (TLR) for LPS is TLR4. Herein, we show that enzymatically-inactive (EI) tPA blocks the response of mouse BMDMs to selective TLR2 and TLR9 agonists, rapidly reversing IκBα phosphorylation and inhibiting expression of TNFα, CCL2, interleukin-1β, and interleukin-6. The activity of EI-tPA was replicated by activated α_2_-macroglobulin, which like EI-tPA, signals through an NMDA-R-dependent pathway. EI-tPA failed to inhibit cytokine expression by BMDMs in response to agonists that target the Pattern Recognition Receptors (PRRs), NOD1 and NOD2, providing evidence for specificity in the function of EI-tPA. Macrophages isolated from the peritoneal space (PMs), without adding eliciting agents, expressed decreased levels of cell-surface NMDA-R compared with BMDMs. These cells were unresponsive to EI-tPA in the presence of LPS. However, when PMs were treated with CSF-1, the abundance of cell-surface NMDA-R increased and the ability of EI-tPA to neutralize the response to LPS was established. We conclude that the anti-inflammatory activity of EI-tPA is selective for TLRs but not all PRRs. The ability of macrophages to respond to EI-tPA depends on the availability of cell surface NMDA-R, which may be macrophage differentiation-state dependent.

## Introduction

In monocytes and macrophages, Pattern Recognition Receptors (PRRs) recognize molecules produced by invading pathogens and activate cell-signaling and gene expression programs associated with innate immunity [1, 2]. PRRs include but are not limited to Toll-like receptors (TLRs), C-type Lectin Receptors (CLRs), and Nucleotide-binding Oligomerization Domain-like receptors (NOD-like receptors). TLR4 is a well-studied TLR, which plays an essential role in the response to lipopolysaccharide (LPS) released by gram-negative bacteria [3–5]. Efficient recognition of LPS by TLR4 requires myeloid differentiation factor 2 (MD2) and proteins involved in LPS delivery to TLR4-MD2 complex, including CD14 and LPS binding protein [6–8]. Complexity in the LPS recognition and delivery system provides multiple opportunities for regulation.

Tissue-type plasminogen activator (tPA) is a serine proteinase and activator of fibrinolysis, which has been used under specific conditions to treat ischemic stroke [9, 10]. tPA also is active in innate immunity, suppressing the response to LPS in mouse bone marrow-derived macrophages (BMDMs) and *in vivo* in mice [11, 12]. The pathway by which tPA modifies the response to LPS is incompletely understood but involves rapid reversal of TLR4-mediated IκBα phosphorylation, which prevents sustained Nuclear Factor Kappa-B (NFκB) activation and cytokine expression [12]. The function of tPA as an LPS response modifier is not dependent on its protease activity and is replicated by enzymatically-inactive tPA (EI-tPA). Instead, the effects of tPA on BMDMs are mediated by the N-methyl-D-aspartate receptor (NMDA-R), which is best known for its function as a neuronal synapse protein but also expressed by macrophages [12, 13]. LDL Receptor-related Protein-1 (LRP1), a transmembrane receptor that binds tPA [14–18], probably functions as an NMDA-R co-receptor, decreasing the concentration of tPA required to trigger NMDA-R-dependent cell-signaling and gene regulatory events [17–20].

The first major objective of the current study was to determine whether tPA functions as an anti-inflammatory agent with agonists and receptors other than LPS and TLR4. To address this question, we studied the effects of EI-tPA and activated α_2_-macroglobulin (α_2_M) on macrophage responses initiated by agonists for TLR2, TLR4, TLR9, NOD1, and NOD2. Studying EI-tPA as opposed to active tPA avoided possible confounding effects resulting from plasminogen activation. We show that in BMDMs, EI-tPA and α_2_M antagonize the activity of multiple TLRs but do not attenuate pro-inflammatory cytokine expression induced by NOD1 or NOD2 agonists. Quiescent macrophages, isolated from the peritoneal space of mice without thioglycollate elicitation (PMs), did not respond to EI-tPA unless these cells were first treated with colony-stimulating factor-1 (CSF-1), which increased the abundance of cell-surface NMDA-R in PMs to the level observed in BMDMs. We conclude that the inhibitory activity of tPA in innate immunity is evident with multiple TLRs but not with PRRs in general. Our results further support an essential role for the NMDA-R as a macrophage receptor that confers responsiveness to tPA.

## Materials and methods

### Proteins and reagents

Human EI-tPA, which carries the S478→A mutation and is 90% in the single-chain form, was from Molecular Innovations. α_2_M was purified from human plasma and activated for binding to LRP1 by reaction with methylamine, as previously described [21]. Recombinant mouse CSF-1 was from R&D Systems. LPS serotype 055:B5 from *E*. *coli* was from Sigma-Aldrich. Lipoteichoic acid (LTA) from *Staphylococcus aureus* was from InvivoGen. LTA is a selective TLR2 agonist, which does not cross-activate other TLRs such as TLR1 or TLR4 [22, 23]. The synthetic un-methylated CpG-containing oligodeoxynucleotide, ODN 1826, which selectively activates TLR9 [24], and C12-iE-DAP, which is an acylated derivative of a peptidoglycan component found in bacteria and a selective NOD1 agonist [25], also were from InvivoGen. Muramyl dipeptide (MDP), the core bacterial peptidoglycan component present in all bacteria and a selective NOD2 agonist [26], was from Sigma-Aldrich. L929 cells were obtained from the ATCC and cultured in DMEM/F12 with 10% FBS for 5 days. Conditioned medium was harvested as described [11].

### Macrophage cultures

Procedures for isolating macrophages from mice were approved by the University of California San Diego Institutional Animal Care and Use Committee. BMDMs were harvested from 16 week-old wild-type male C57BL/6J mice (The Jackson Laboratory, #000664), as previously described [11]. Mice were euthanized by CO_2_ asphyxiation, followed by cervical dislocation. Bone marrow cells were flushed from mouse femurs and plated in non-tissue culture-treated dishes in DMEM/F-12 medium containing 10% fetal bovine serum (FBS) and 20% L929 cell-conditioned medium for 8 days. Non-adherent cells were eliminated. Adherent cells included >95% BMDMs, as determined by F4/80 and CD11b immunoreactivity. Because this harvesting procedure was performed following euthanasia, additional approaches to alleviate suffering were not necessary.

Quiescent PMs were isolated without thioglycollate elicitation and cultured as previously described [27]. In brief, 5 month old male C57BL/6J mice were euthanized, as described above. Using a 25-gauge needle, 20 mM sodium phosphate, 150 mM NaCl, pH 7.4 (PBS) with 3% FBS and 1% penicillin/streptomycin (pen/ strep) (5 mL) was injected into the peritoneal space. The solution was massaged from the abdominal surface and then harvested using the same needle. The procedure was repeated three times. Isolates that contained visible red blood cells were excluded. The remaining isolates were subjected to centrifugation at 800 × g for 5 min, suspended in DMEM/F12 supplemented with 10% FBS and 1% pen/strep, and then plated at 2 x 10^6^ cells/well in tissue-culture treated, 6-well plates. The cells were washed extensively 2 h after plating. PMs were maintained in culture for up to 5 days before conducting experiments and then studied as a single population without attempting to sort these cells based on size. In some studies, PMs were treated with CSF-1 (10 ng/mL) or with 20% L929 cell-conditioned medium, which is a source of CSF-1 [28], for up to 5 days.

Before conducting experiments to examine cytokine expression, BMDMs and PMs were transferred to serum-free medium (SFM) for 30 min and then treated with various proteins and reagents, alone or simultaneously as noted, including: LTA (10–100 ng/mL); ODN 1826 (0.1–1 μM); LPS (0.1 ng/ml) C12-iE-DAP (1 μg/mL); MDP (50 μg/mL); EI-tPA (12 nM); activated α_2_M (10 nM); or vehicle (PBS).

### RT-qPCR analysis of cytokine mRNA expression

RNA was isolated using the NucleoSpin RNA kit (Macherey-Nagel) and reverse-transcribed using the iScript cDNA synthesis kit (Bio-Rad). RT-qPCR was performed to determine the relative abundance of mRNAs encoding tumor necrosis factor-α (TNFα), interleukin-1β (IL-1β), interleukin-6 (IL-6), and C-C Motif Chemokine Ligand 2 (CCL2) using TaqMan gene expression products (ThermoFisher). Relative changes in mRNA expression were calculated using the 2^ΔΔCt^ method and GAPDH mRNA as an internal normalizer.

### Analysis of cell-signaling

BMDMs were transferred to SFM for 30 min and then treated as indicated. Cell extracts were prepared in RIPA buffer containing Protease Inhibitors and Phosphatase Inhibitors Cocktail (Pierce). Protein concentrations were determined by DC Protein Assay (Bio-Rad). Equivalent amounts of cellular protein were subjected to SDS PAGE and electrotransferred to polyvinylidene difluoride membranes. Membranes were blocked and probed with primary antibodies from Cell Signaling Technology that target: phospho-IκBα (Ser32) (1:1000, cat. 2859), total IκBα (1:1000, 9242), and β-actin (1:5000, 3700), followed by horseradish peroxidase-conjugated secondary antibodies. Immunoblots were developed using ProSignal Pico ECL Reagent substrate (Prometheus) and imaged using the Azure Biosystems c300 imaging system.

### Flow cytometry

PMs were cultured in DMEM/F12 with 10% FBS alone or in the presence of 20% L929 cell-conditioned medium or 10 nM CSF-1 for up to 5 days. PMs and BMDMs were suspended using 0.5% Trypsin (Invitrogen). Non-specific antibody binding was blocked by incubation with 2% BSA in PBS. The cells were then treated with NMDA-R NR1 subunit-specific antibody (Invitrogen, cat. PA3-102), diluted 1:100 for 40 min. After washing to remove unbound antibody, the cells were incubated with Alexa Fluor 647-conjugated anti-rabbit secondary antibody (ThermoFisher, 1:600) for 30 min. Unbound antibody was removed by washing. All incubations were performed with live cells at 4°C so that antibody-binding was limited to cell surface epitopes. At least 10,000 cells per sample were analyzed using a BD FACS Canto II (BD Biosciences). Flow histogram overlays were prepared using FlowJo software (FlowJo, LLC).

### Immunofluorescence (IF) microscopy

PMs were cultured on glass slides for up to 5 days in the presence of 10 nM CSF-1 or vehicle. The cells were no more than 70% confluent at the end of the incubations. The PMs were then incubated with NMDA-R NR1 antibody (Invitrogen, cat. PA3-102) diluted 1:100 for 40 min at 4°C. After washing, the cells were incubated with anti-rabbit Alexa Fluor 488 conjugated secondary antibody (ThermoFisher, 1:600) for 30 min at 4°C. PMs were fixed in 2% formaldehyde for 10 min, washed with distilled water and mounted on slides using DAPI-containing Prolong Diamond Antifade (Invitrogen). Images were acquired using Leica DMi8 Inverted Fluorescence microscope (Leica Microsystems) equipped with Leica DFC3000 G camera and analyzed by ImageJ.

### Statistics

Statistical analysis was performed using GraphPad Prism 5.0. Results are presented as the mean ± SEM. Each replicate was performed using macrophage cultures from a separate mouse. Data were analyzed by one-way ANOVA followed by Bonferroni post-hoc test on selected pairs of columns (*p<0.05, **p<0.01, ***p<0.001).

## Results

### EI-tPA blocks the response of BMDMs to TLR2 agonist

**Fig 1:**
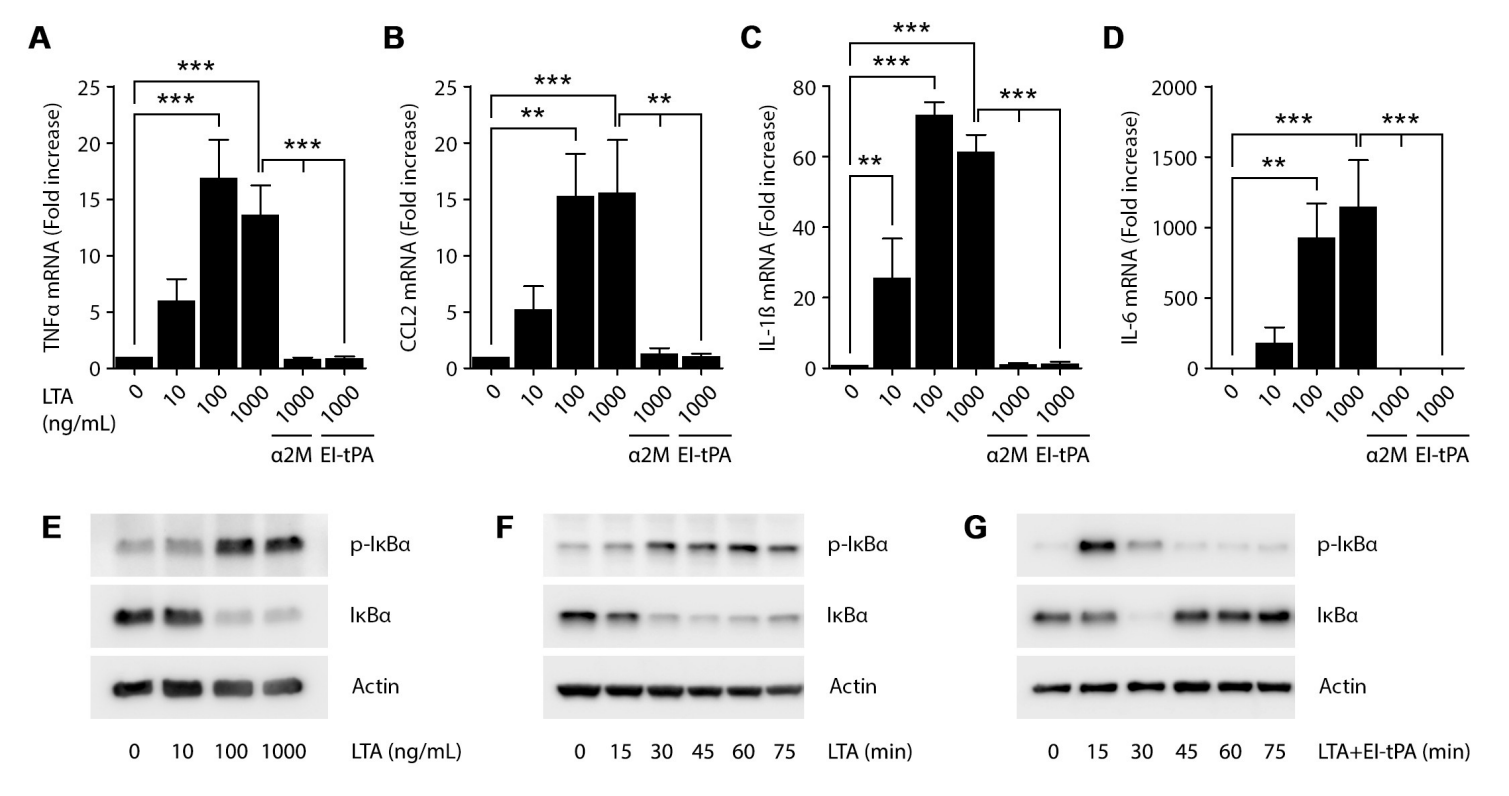
EI-tPA blocks TLR2-induced inflammatory response in BMDMs. BMDMs were treated with increasing concentrations of the TLR2 agonist, LTA (0.01–1 μg/mL), or with LTA (1 μg/mL) plus activated α2M (10 nM) or EI-tPA (12 nM) for 6 h. RT-qPCR was performed to compare mRNA levels of (A) TNFα, (B) CCL2, (C) IL-1β, and (D) IL-6 (mean ± SEM; n = 4–5; one-way ANOVA with Bonferroni post-hoc test on selected pairs of columns; **p<0.01, ***p<0.001). Immunoblot analysis showing total and phosphorylated IκBα in BMDMs treated with: (E) increasing concentrations of LTA (0.01–1 μg/mL) for 1 h; (F) 1 μg/mL LTA for the indicated times; and (G) 1 μg/mL LTA plus 12 nM EI-tPA for the indicated times. β-actin levels are shown as a control.

LTA is a selective TLR2 agonist, produced by gram-positive bacteria and isolated from the bacterial envelope [22, 23, 29]. When BMDMs were treated with 0.1–1.0 μg/mL LTA for 6 h, TNFα mRNA expression was significantly increased (Fig 1A). When BMDMs were treated with 1.0 μg/mL LTA in the presence of 12 nM EI-tPA, the LTA-induced increase in TNFα mRNA expression was blocked. Activated α_2_M (10 nM) also blocked the effects of LTA on TNFα mRNA. In addition to TNFα, LTA increased expression of the mRNAs encoding CCL2, IL-1β, and IL-6 (Fig 1B–1D). EI-tPA and activated α_2_M both blocked the effects of LTA on expression of each of these pro-inflammatory cytokines.

IκBα binds NFκB in the cytoplasm and prevents NFκB translocation into the nucleus [30, 31]. When IκBα is phosphorylated, NFκB dissociates and transfers to the nucleus where it regulates gene transcription. Although phosphorylated IκBα is rapidly degraded, new IκBα is expressed under the control of activated NFκB, replenishing IκBα protein in the cell [32]. In BMDMs, LPS induces sustained IκBα phosphorylation [12]. The total level of IκBα decreases substantially at first, due to degradation of phosphorylated IκBα; however, the total level of IκBα protein partially recovers in an apparently cyclical manner, probably reflecting a balance between increased IκBα protein expression and degradation of phosphorylated IκBα [12]. In BMDMs treated with LPS and EI-tPA simultaneously, IκBα is initially phosphorylated and total level of IκBα is decreased; however, these events are rapidly reversed and the transient increase in phosphorylated IκBα is insufficient to drive inflammatory cytokine expression [12].

In BMDMs treated with 0.1 or 1.0 μg/mL LTA for 60 min, IκBα was phosphorylated and the total abundance of IκBα protein was decreased, indicating NFκB activation, as anticipated (Fig 1E). In time course experiments, BMDMs treated with 1.0 μg/mL LTA demonstrated increased phosphorylated IκBα by 15–30 min and the increase in phosphorylated IκBα was sustained throughout the 75 min experiment. (Fig 1F). The increase in phosphorylated IκBα was accompanied by a decrease in the total abundance of IκBα, which also was sustained through 75 min.

In BMDMs treated simultaneously with 1.0 μg/mL LTA and 12 nM EI-tPA, IκBα was phosphorylated by 15 min; however, unlike in cells treated with LTA alone, the level of phosphorylated IκBα returned to pre-treatment levels by 45 min (Fig 1G). The total abundance of IκBα protein decreased transiently, but also returned to pre-treatment levels by 45 min. These results demonstrate that EI-tPA does not prevent IκBα phosphorylation and the initial decrease in total abundance of IκBα in response to LTA but instead, rapidly reverses this response, mimicking the events observed in cells treated with LPS [12].

### EI-tPA blocks the response of BMDMs to TLR9 agonist

**Fig 2:**
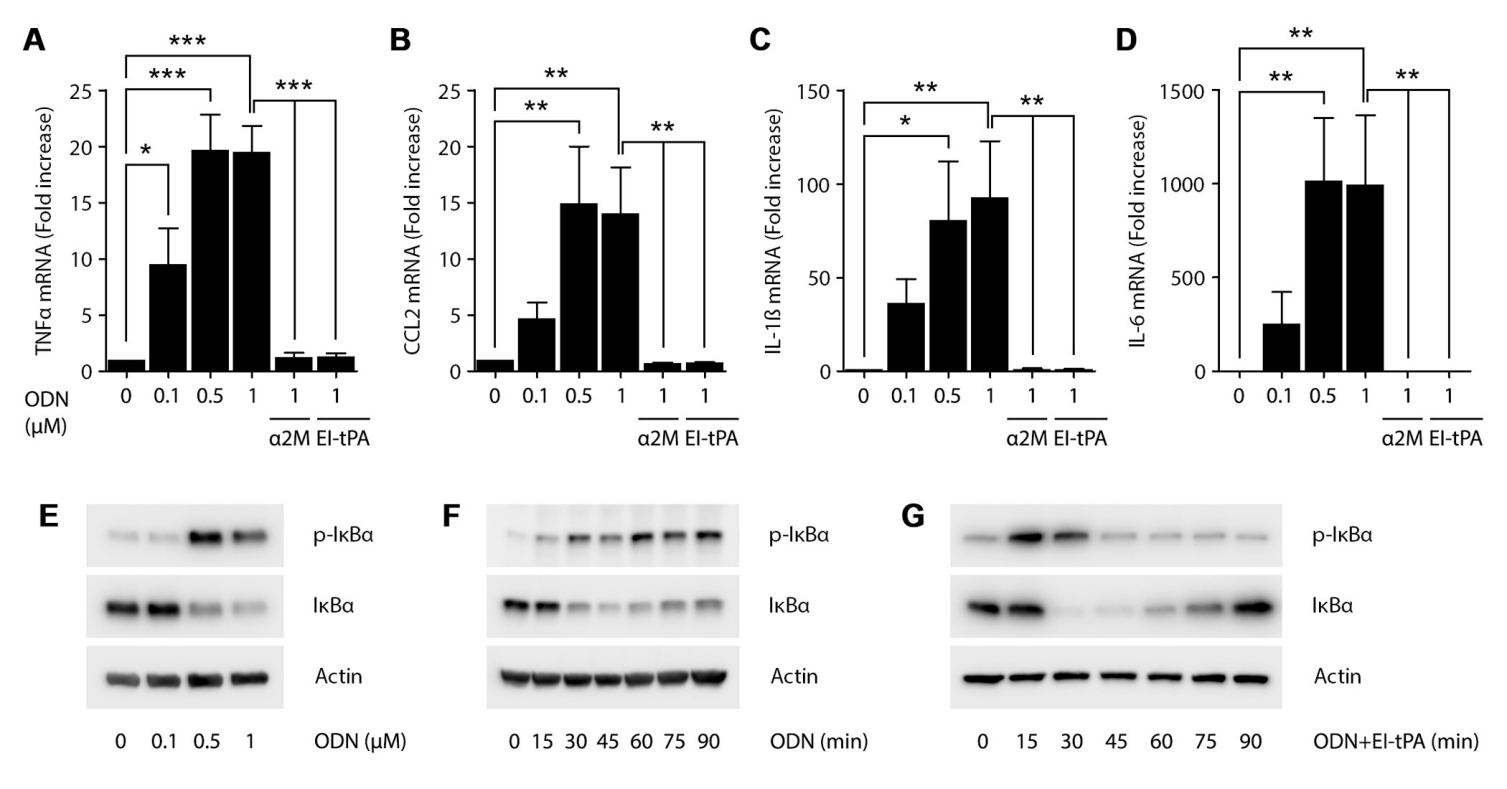
EI-tPA blocks TLR9-induced inflammatory response in BMDMs. BMDMs were treated with increasing concentrations of the TLR9 agonist, ODN 1826 (0.1–1 μM), or with ODN 1826 (1 μM) plus activated α2M (10 nM) or EI-tPA (12 nM) for 6 h. RT-qPCR was performed to compare mRNA levels of (A) TNFα, (B) CCL2, (C) IL-1β, and (D) IL-6 (mean ± SEM; n = 4–6; one-way ANOVA with Bonferroni post-hoc test on selected pairs of columns; *p<0.05, **p<0.01, ***p<0.001). Immunoblot analysis showing total and phosphorylated IκBα in BMDMs treated with: (E) increasing concentrations of ODN 1826 (0.1-1 μM) for 1 h; (F) 1 μM ODN 1826 for the indicated times; or (G) 1 μM ODN 1826 in presence of 12 nM EI-tPA for the indicated times. β-actin levels are shown as control.

TLR9 functions largely in endosomes to recognize un-methylated CpG sequences, which are more prevalent in viral and bacterial DNA compared with eukaryotic DNA [24, 33]. Treatment of BMDMs with the TLR9 agonist, ODN 1826, at concentrations of 0.1–1.0 μM, significantly increased expression of TNFα mRNA (Fig 2A). Simultaneous treatment of cells with 1.0 μM ODN 1826 and either 12 nM EI-tPA or 10 nM activated α_2_M blocked the increase in TNFα mRNA observed with ODN 1826 alone.

In addition to TNFα, BMDMs treated with ODN 1826 (0.5–1.0 μM) expressed increased levels of the mRNAs encoding CCL2, IL-1β, and IL-6 (Fig 2B–2D). In each case, EI-tPA neutralized the increase in expression of the pro-inflammatory cytokine caused by ODN 1826. Activated α_2_M demonstrated equivalent activity.

IκBα was phosphorylated in BMDMs treated for 1 h with 0.5 or 1.0 μM ODN 1826 (Fig 2E). In time course experiments, in which BMDMs were treated with 1.0 μM ODN 1826, the increase in phosphorylated IκBα was apparent by 15–30 min and sustained throughout the 90 min experiment (Fig 2F). In the same timeframe, the total abundance of IκBα decreased although low levels of recovery were observed at 75 and 90 min in some replicates, including the experiment that is shown. When BMDMs were treated simultaneously with 1.0 μM ODN 1826 and 12 nM EI-tPA, IκBα phosphorylation was observed at 15–30 min; however, by 45 min, phosphorylated IκBα decreased sharply to pre-treatment levels (Fig 2G). The total abundance of IκBα initially declined but recovered completely by 90 min. Thus, in the presence of ODN 1826, EI-tPA prevents sustained IκBα phosphorylation and promotes recovery of total IκBα protein, mimicking the effects of EI-tPA on the LPS response, which were previously shown to be linked to the ability of EI-tPA to prevent expression of pro-inflammatory cytokines [12]. The results presented here and previously [12] demonstrate that EI-tPA has a similar effect on NFκB activation in response to agonists for TLR2, TLR9, and TLR4.

### EI-tPA is ineffective at neutralizing NOD1 and NOD2

NOD1 and NOD2 are cytoplasmic proteins that, like TLRs, function in innate immunity by mediating inflammatory responses to foreign molecules [34, 35]. Chronic NOD2 stimulation may induce immune tolerance to bacterial products [36]. Mutations in NOD2 are associated with Crohn’s Disease [37, 38].

**Fig 3:**
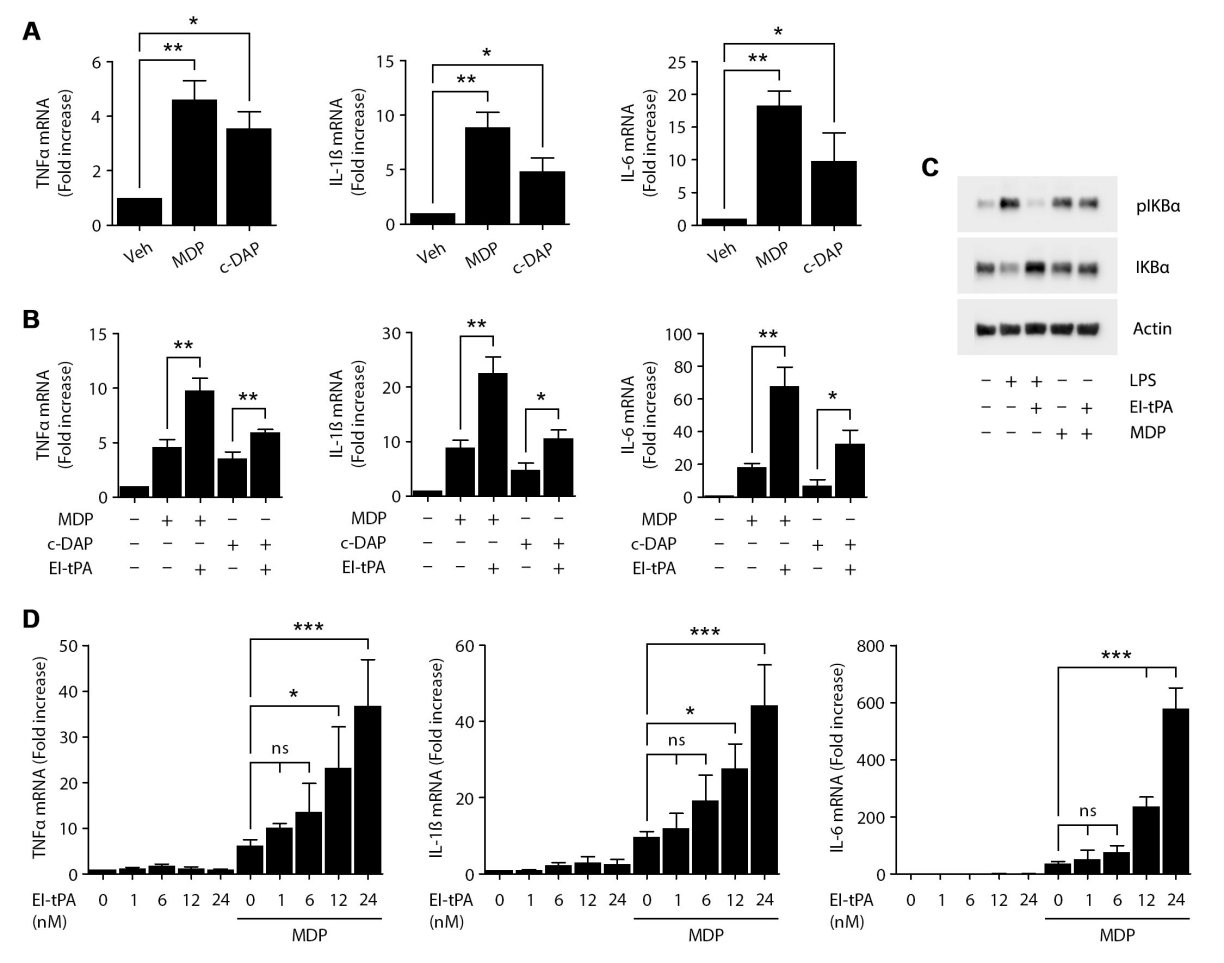
EI-tPA does not antagonize pro-inflammatory cytokine expression in response to NOD1/2 agonists. RT-qPCR was performed to determine mRNA expression of TNFα, IL-1β and IL-6 in: (A) BMDMs treated with the NOD1 agonist, C12-iE-DAP (c-DAP) (1 μg/ml), the NOD2 agonist, MDP (50 μg/ml), or vehicle for 6 h; and in (B) BMDMs treated with MDP or C12-iE-DAP in the presence or absence of 12 nM EI-tPA for 6 h (mean ± SEM, n = 4; one-way ANOVA with Bonferroni post-hoc test; *p<0.05, **p<0.01). (C) BMDMs were treated for 1 h with LPS (0.1 μg/mL), MDP (50 μg/ml), or vehicle in the presence or absence of 12 nM EI-tPA. Immunoblot analysis was performed to determine phosphorylated IκBα, total IκBα and β-actin. (D) BMDMs were treated with increasing concentrations of EI-tPA (0–24 nM), alone or in the presence of 50 μg/ml of MDP for 6 h. RT-qPCR was performed to determine expression of TNFα, IL-1β and IL-6 (mean ± SEM, n = 4; one-way ANOVA with Bonferroni post hoc test on selected pairs of columns *p<0.05, ***p<0.001, ns = not significant).

We treated BMDMs with the NOD1 agonist, C12-iE-DAP (1 μg/mL), or with the NOD2 agonist, MDP (50 μg/mL) for 6 h. Fig 3A shows that the mRNAs encoding TNFα, IL-1β, and IL-6 were increased by both agonists. When BMDMs were treated with MDP and 12 nM EI-tPA simultaneously, EI-tPA failed to neutralize the effects of MDP on cytokine expression. In fact, statistically significant increases in expression of TNFα, IL-1β, and IL-6 were observed, compared with that detected in cells treated with MDP alone (Fig 3B). Similar results were obtained with C12-iE-DAP. Increased pro-inflammatory cytokine expression was detected in BMDMs treated with C12-iE-DAP. Simultaneous addition of EI-tPA failed to neutralize the response to C12-iE-DAP and instead, significantly increased cytokine expression compared with the levels observed in cells treated with C12-iE-DAP alone.

Next, we examined the effects of MDP on IκBα phosphorylation and the total abundance of IκBα protein in BMDMs, in the presence and absence of EI-tPA. As a control, we also treated BMDMs with LPS. In BMDMs treated with LPS (0.1 μg/mL), in the absence of EI-tPA, for 1 h, IκBα was phosphorylated and the total abundance of IκBα protein was decreased, as anticipated (Fig 3C). EI-tPA blocked the increase in IκBα phosphorylation and the decrease in the total abundance of IκBα protein caused by LPS, confirming our earlier results [12]. In the absence of EI-tPA, MDP also caused IκBα phosphorylation. The total abundance of IκBα was not substantially altered, which probably reflects our sampling of a single time point. Importantly, EI-tPA failed to reverse the increase in IκBα phosphorylation caused by MDP.

Because we did not anticipate that 12 nM EI-tPA would significantly augment the response of BMDMs to MDP, we conducted EI-tPA dose-dependency experiments in cells treated simultaneously with 50 μg/mL MDP. Fig 3D shows that EI-tPA dose-dependently increased expression of the mRNAs encoding TNFα, IL-1β, and IL-6. Although these results were not explained mechanistically in the present study, EI-tPA is known to independently regulate phosphorylation of multiple cell-signaling proteins in BMDMs, including ERK1/2, Akt, and Chk2 [12]. These tPA-induced signaling events may amplify the response to MDP without having a similar effect on TLR-mediated responses.

### The activity of EI-tPA as a TLR response modifier is macrophage differentiation state-dependent

**Fig 4:**
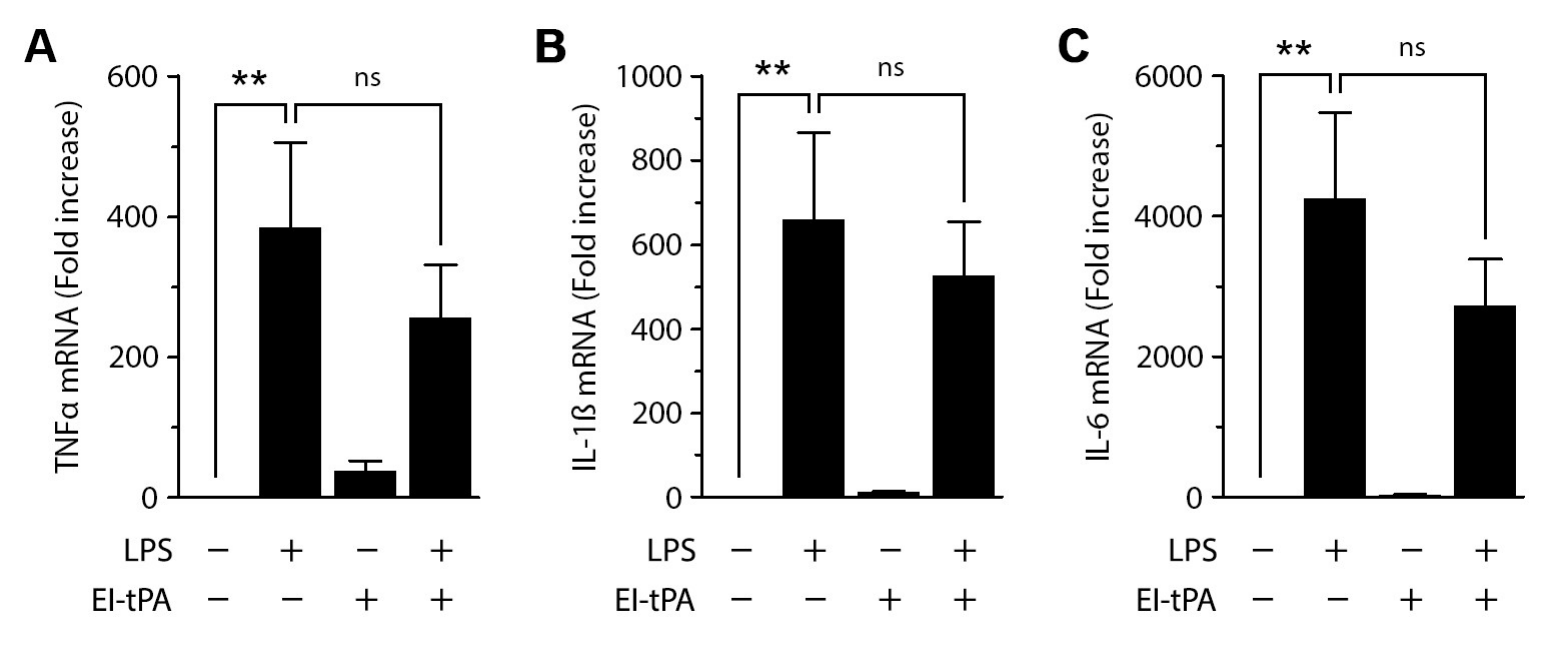
EI-tPA does not inhibit LPS-induced pro-inflammatory cytokine expression in PMs. Quiescent PMs were treated with LPS (0.1 μg/mL), EI-tPA (12 nM), LPS plus EI-tPA, or vehicle for 6 h. RT-qPCR was performed to determine expression of the mRNAs encoding: (A) TNFα; (B) IL-1β; and (C) IL-6 (mean ± SEM, n = 4; one-way ANOVA with Bonferroni post-hoc test, **p<0.01, ns = not significant).

In our standard procedure for preparing mouse BMDMs, the cells are differentiated by exposure to L929 cell conditioned medium, a source of CSF-1 [28]. To examine the activity of EI-tPA as a TLR response modifier in macrophages that are not pre-exposed to differentiating or activating agents, we isolated mouse peritoneal macrophages (PMs) without introducing eliciting agents into the peritoneal space. When treated with LPS *in vitro*, PMs expressed increased levels of the mRNAs encoding TNFα, IL-1β, and IL-6 as anticipated (Fig 4A–4C). EI-tPA (12 nM) did not regulate expression of pro-inflammatory cytokines in PMs in the absence of LPS; however, EI-tPA also failed to significantly attenuate the response to LPS.

**Fig 5:**
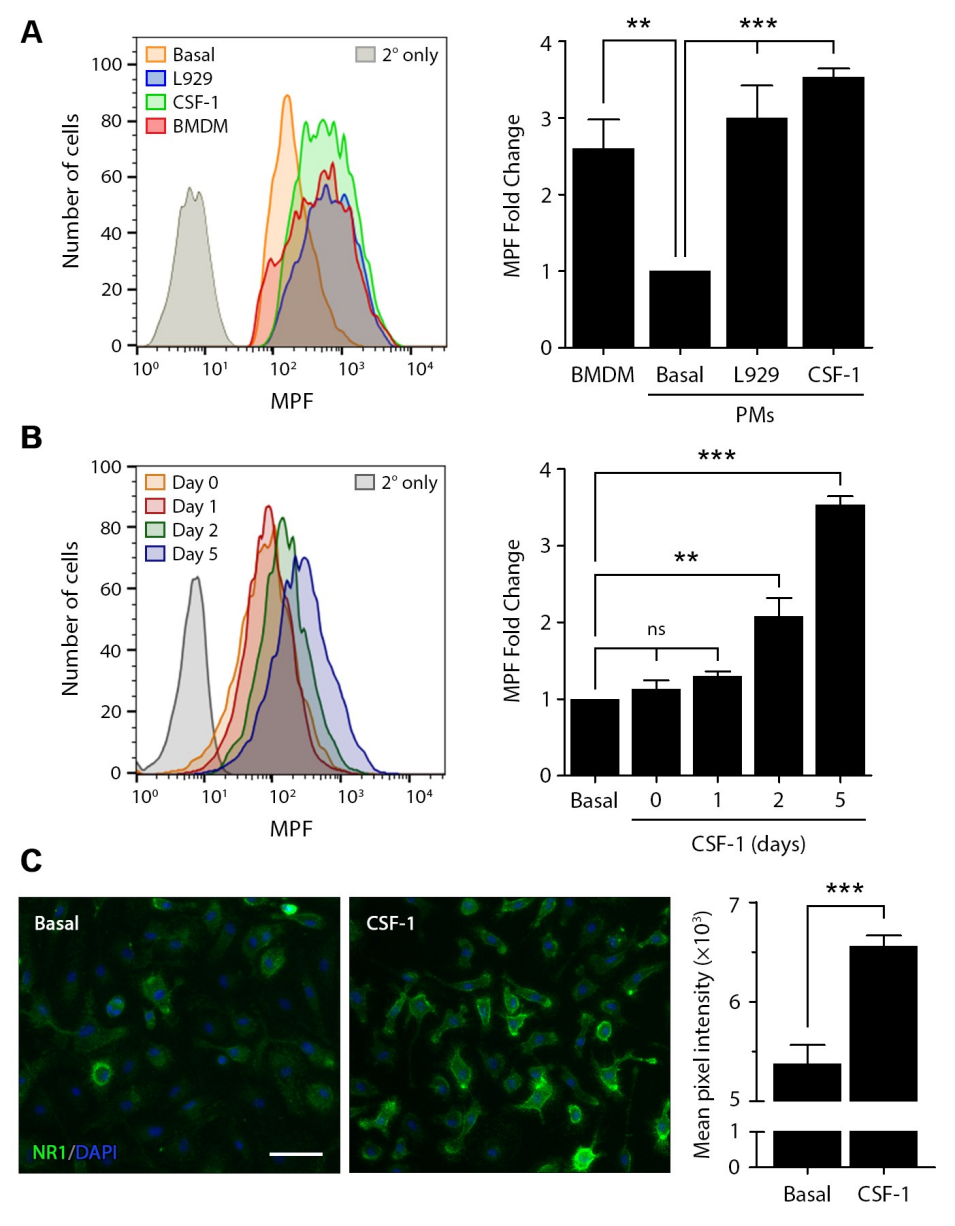
CSF-1 regulates cell surface NMDA-R in macrophages. Cell surface NMDA-R was labeled in BMDMs and PMs using NR1 subunit-specific primary antibody and Alexa Fluor 647-conjugated secondary antibody. (A) Representative flow cytometry histograms are shown for BMDMs, quiescent PMs (basal), PMs treated with L929 cell-conditioned medium for 5 days (L929), and PMs treated with CSF-1 (10 ng/mL) for 5 days. As a control, PMs were treated with secondary antibody alone (2° only). The bar graph (right panel) shows the fold increase in the MPF compared with quiescent PMs (no exposure to L929 cell conditioned medium or CSF-1) (mean ± SEM, n = 4; one-way ANOVA with Bonferroni post-hoc test, **p<0.01, ***p<0.001). (B) Representative NMDA-R NR1 subunit flow cytometry histograms are shown for PMs treated with CSF-1 (10 ng/mL) for increasing periods of time (0, 1, 2, and 5 days). The fold increase in the MPF compared to untreated cells is presented (mean ± SEM, n = 4; one-way ANOVA with Bonferroni post-hoc test, **p<0.01, ***p<0.001, ns = not significant). (C) IF microscopy images showing NMDA-R NR1 subunit in fixed, non-permeabilized PMs cultured in basal medium (left) or in CSF-1-supplemented medium (right) for 2 days. Images are representative of 3 independent experiments (scale bar, 10 μm). The bar graph shows the quantification of mean pixel intensity as measured in 14 independent fields of view in three separate slides (mean ± SEM, unpaired t test, ***p<0.001).

Because the ability of EI-tPA to function as an LPS response modifier in BMDMs is dependent on the NMDA-R [12], we performed flow cytometry experiments to compare levels of cell surface NMDA-R NR1 subunit in BMDMs and PMs. The NR1 subunit is present on the cell surface only when it is a component of intact NMDA-R channels [39, 40]. Fig 5A shows that, by flow cytometry, the abundance of cell surface NR1, as determined by mean peak fluorescence (MPF), was more than 2.5-fold higher in BMDMs compared with PMs. When PMs were exposed to 20% L929 cell conditioned medium or to purified recombinant CSF-1 (10 ng/mL) for 5 days, the MPF increased to approximately the level observed in BMDMs. The increase in abundance of cell-surface NMDA-R, observed in CSF-1-treated PMs, was time-dependent (Fig 5B). A statistically significant increase in MPF was detected at 48 h and a further increase in MPF was apparent by 5 days.

We confirmed that cell-surface NMDA-R is increased in PMs 48 h after introducing CSF-1 by IF microscopy (Fig 5C). In these experiments, NR1-specific antibody was incubated with non-permeabilized, viable PMs at 4°C to selectively detect cell-surface NMDA-R. In the absence of CSF-1, cell-surface NMDA-R was detected at low levels. After treating PMs with CSF-1, a substantial increase in NMDA-R NR1 immunofluorescence was observed. Image analysis of the cells in 14 separate high power fields from three different experiments confirmed that the mean NR1 fluorescence intensity was significantly increased in PMs following CSF-1 treatment (p<0.001).

**Fig 6:**
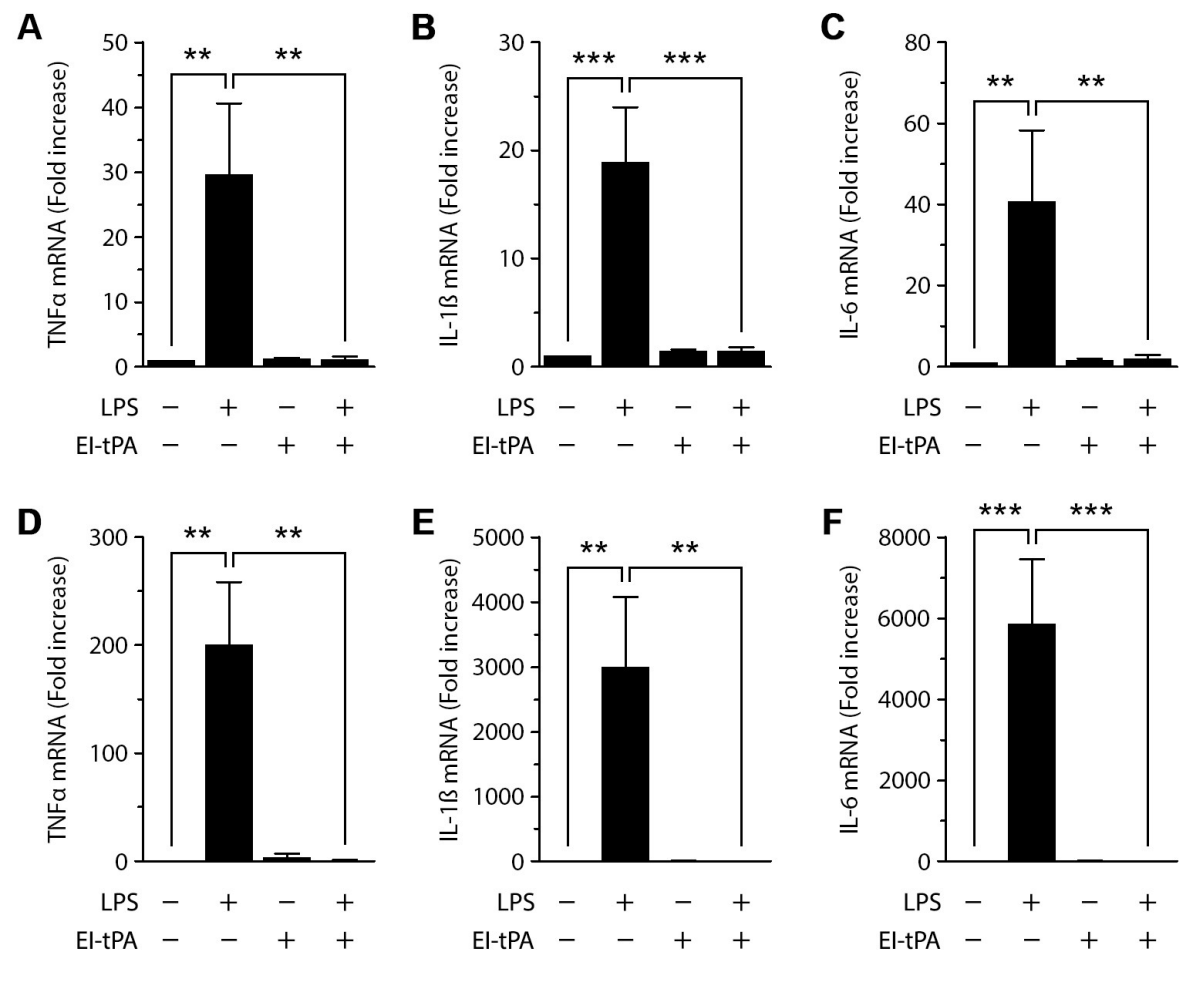
EI-tPA blocks the response to LPS in CSF-1-differentiated PMs by an NMDA-R-dependent pathway. PMs were cultured in basal media supplemented with L929 cell-conditioned medium (panels A-C) or CSF-1 (10 ng/mL) (panels D-F) for 48 h and then treated with LPS (0.1 μg/mL), EI-tPA (12 nM), LPS plus EI-tPA, or vehicle for 6 h. Expression of TNFα (panels A, D), IL-1β (panels B, E) and IL-6 (panels C, F) was determined by RT-qPCR (mean ± SEM, n = 3–4; one-way ANOVA with Bonferroni post-hoc test on selected pairs of columns, **p<0.01, ***p<0.001).

To test whether the increase in abundance of cell-surface NMDA-R caused by PM differentiation induced responsiveness to EI-tPA, first PMs were treated with L929 cell-conditioned medium for 48 h and then with LPS (0.1 μg/mL). In the absence of EI-tPA, LPS significantly increased expression of the mRNAs encoding TNFα, IL-1β, and IL-6 (Fig 6A–6C). The magnitude of the increase in cytokine expression caused by LPS was somewhat lower in L929 cell-conditioned medium-treated PMs compared with undifferentiated PMs. This result did not reflect a difference in the basal level of expression of cytokines, prior to LPS treatment, but instead, a change in the ability of the cells to respond to LPS. In the absence of LPS, EI-tPA alone had no effect on pro-inflammatory cytokine expression in L929 cell-conditioned medium pre-treated PMs; however, importantly, in these cells, EI-tPA blocked cytokine expression in response to LPS.

Next, we studied PMs that were differentiated with purified CSF-1. In these cells, the LPS response was more robust in the absence of EI-tPA, mimicking the response observed in undifferentiated PMs (Fig 6D–6F). Once again in these PMs, EI-tPA did not independently regulate expression of pro-inflammatory cytokines; however, when CSF-1-pre-treated PMs were treated with LPS and EI-tPA simultaneously, EI-tPA completely blocked the LPS response.

## Discussion

Herein, we demonstrate for the first time that EI-tPA and activated α_2_M effectively neutralize TLR-dependent responses in BMDMs that are independent of TLR4 and the LPS recognition system. LTA binds to TLR2, which initiates cell-signaling from the cell surface like TLR4. TLR2 functions as a homodimer or as a heterodimer with either TLR1 or TLR6 [41]. ODN 1826 binds to TLR9, which activates cell-signaling from within endosomes [42]. Given that EI-tPA and α_2_M neutralize more than one TLR, including TLRs at the cell surface and within endosomes, our results support a model in which regulation of TLR activity occurs intracellularly, at the level of cell-signaling. We propose that cell signaling factors, activated downstream of the NMDA-R/LRP1 signaling receptor complex, are responsible for the rapid reversal of IκBα phosphorylation observed in this study, in experiments with agonists for TLR2 and TLR9, and with LPS previously [12]. TLR4, TLR2, and TLR9 all signal through a MyD88-dependent pathway, which culminates in activation of NFκB [3]. The signaling proteins in this pathway are candidate targets for the NMDA-R/LRP1 receptor complex when this complex is activated by EI-tPA or α_2_M in macrophages. For example, in neurons and neuron-like cells, tPA and α_2_M activate Src family of kinases (SFKs) and phosphatidylinositol 3-kinase (PI3K) in an NMDA-R-dependent manner [43]. The SFK, Lyn, and PI3K are reported to negatively regulate the activity of TLR2 and TLR4 [44].

The inability of EI-tPA to inhibit pro-inflammatory cytokine expression in response to agonists for NOD1 and NOD2, reported here, and our previous data suggesting that EI-tPA does not inhibit expression of pro-inflammatory cytokines in response to activation of PARs [13], suggests that EI-tPA is not a general anti-inflammatory agent but instead, a selective TLR antagonist. This specificity in the function of EI-tPA is important to understand; however, in many human diseases in which TLRs play an active role, other PRRs also are implicated. For example, genetic studies have implicated TLRs in Crohn’s Disease and chronic ulcerative colitis [45], in addition to the well described role of NOD-2 [37, 38]. Furthermore, TLRs interact extensively with NOD-like receptors in innate immunity [46]. Thus, it will be important to assess the activity of EI-tPA in animal models of disease in which inflammation is regulated by diverse PRRs.

In BMDMs treated with NOD1 agonist or NOD2 agonist, simultaneous addition of EI-tPA significantly increased cytokine expression although the effect was modest. We have not explained this result at the molecular level; however, we previously performed phospho-protein arrays to examine cell signaling in BMDMs treated with EI-tPA in the absence of other reagents in an unbiased manner [12]. A number of key cell-signaling factors were regulated by EI-tPA including, for example, ERK1/2, which is reported to be important in promoting pro-inflammatory cytokine expression in macrophages under some circumstances [47]. It is thus possible that EI-tPA-initiated cell-signaling augments NOD1/2 signaling in an additive way, which is not operational when TLRs are activated. Testing this hypothesis will require further work.

The results presented here support our model in which the NMDA-R plays an essential role in the pathway by which EI-tPA regulates gene expression and neutralizes TLR responses in BMDMs. Expression of the NMDA-R and its function in macrophages is not well studied. Shang et al. [48] reported that rat alveolar macrophages express NMDA-R and that the NMDA-R may regulate nitric oxide production. Homocysteine is reported to induce cyclooxygenase-2 expression in macrophages by a pathway that requires the NMDA-R [49]. To study the NMDA-R in BMDMs and PMs, we applied methods that specifically detect the intact hetero-tetramer on the cell surface. In quiescent PMs, the abundance of cell surface NMDA-R was significantly lower than in BMDMs; however, CSF-1 treatment increased cell-surface NMDA-R in PMs. Thus, the level of functional NMDA-R on the surfaces of macrophages appears to be controlled by the macrophage differentiation state.

Importantly, the increase in cell surface NMDA-R induced in PMs by CSF-1 treatment correlated with development of responsiveness to EI-tPA. Macrophages isolated from the peritoneal space responded to EI-tPA only after these cells were treated with purified CSF-1 or with L929 cell-conditioned medium, which is a rich source of CSF-1 used in the preparation of cultured BMDMs [11]. Local and systemic levels of CSF-1 are increased in inflammation and, under these conditions, CSF-1 regulates macrophage gene expression, growth, migration, and differentiation [50, 51]. At this time, we have not determined the mechanism by which CSF-1 controls the cell surface abundance of NMDA-R. Expression of NR1 or any of the NR2 gene products may be involved as may effects of CSF-1 on the subcellular distribution of intact NMDA-R channels. Nevertheless, our results demonstrate that in macrophages, whether the NMDA-R/LRP1 signaling receptor system has the ability to control TLR activity may be regulated by mediators that affect the abundance of the NMDA-R on the cell surface. Interestingly, although macrophages express LRP1 under all conditions, the abundance of cell surface LRP1 is increased by CSF-1 [52]. Thus, CSF-1 up-regulates both known components of the receptor system involved in the macrophage response to EI-tPA and activated α_2_M.

Taken together, the results presented here define two levels of specificity in the function of EI-tPA as a macrophage response modifier. First, EI-tPA specifically antagonizes responses triggered by ligands for TLRs and not PRRs in general. Second, the activity of EI-tPA may be restricted to specific categories of macrophages.

## Supporting information

S1 Raw images Complete, uncropped blots are provided, corresponding to the images presented in the figures of this paper. (PDF)
