# Supplementary material for: Tissue-type plasminogen activator selectively inhibits multiple toll-like receptors in CSF-1-differentiated macrophages
Source: PLoS One. 2019 Nov 7;14(11):e0224738. doi: 10.1371/journal.pone.0224738 (PMC6837328; doi:10.1371/journal.pone.0224738)

Figure 1, panel E

Digital acquisition: Azure c300 Imaging System

phopsho-IκBα (Ser32)

40 kDa, 1:1000

Cell Signaling Technology, #2859

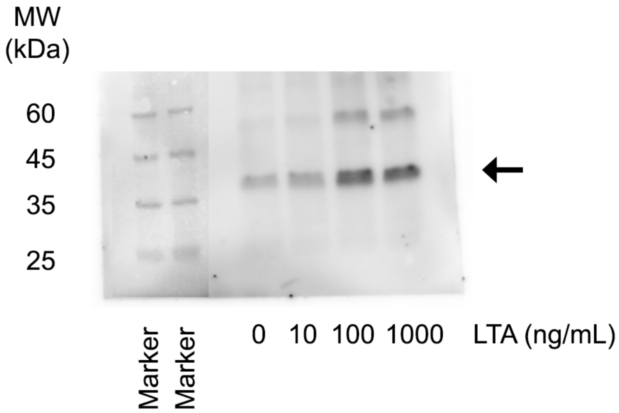

IκBα

39 kDa, 1:1000

Cell Signaling Technology, #9242

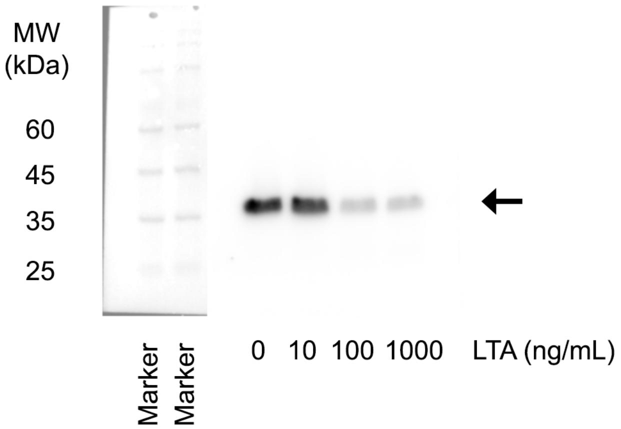

ß-Actin

45 kDa, 1:5000

Cell Signaling Technology, #3700

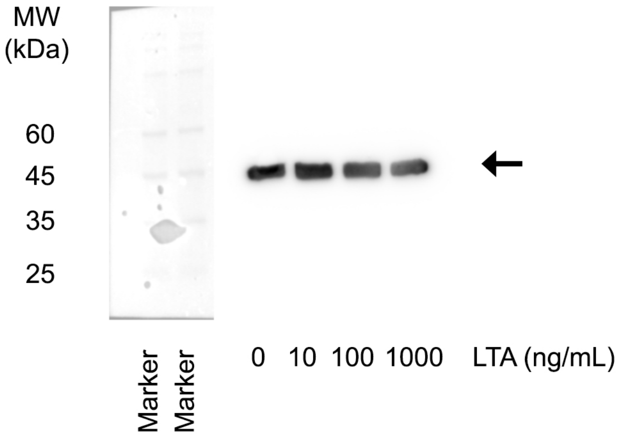

Figure 1, panel F

Digital acquisition: Azure c300 Imaging System

phopsho-IκBα (Ser32)

40 kDa, 1:1000

Cell Signaling Technology, #2859

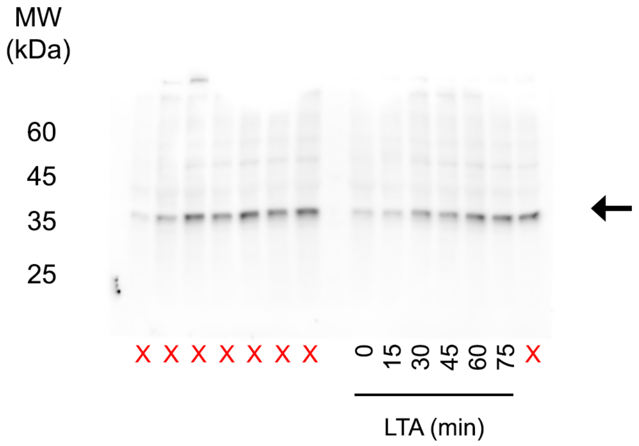

IκBα

39 kDa, 1:1000

Cell Signaling Technology, #9242

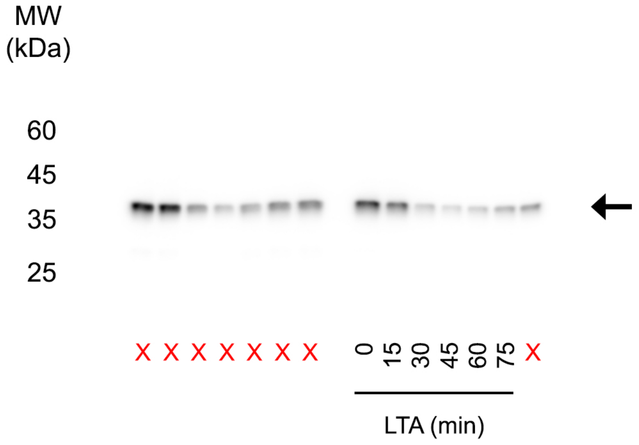

ß-Actin

45 kDa, 1:5000

Cell Signaling Technology, #3700

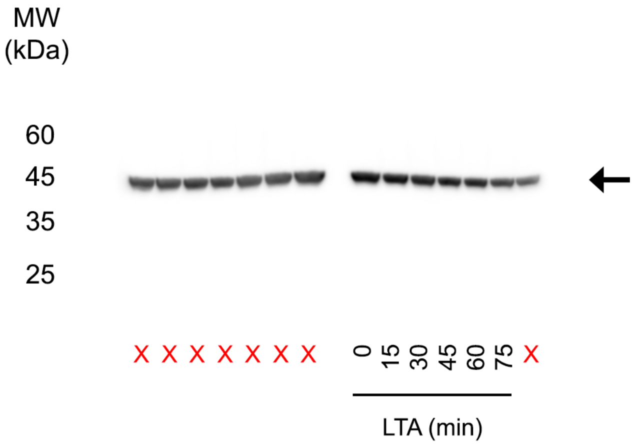

Figure 1, panel G

Digital acquisition: Azure c300 Imaging System

phopsho-IκBα (Ser32)

40 kDa, 1:1000

Cell Signaling Technology, #2859

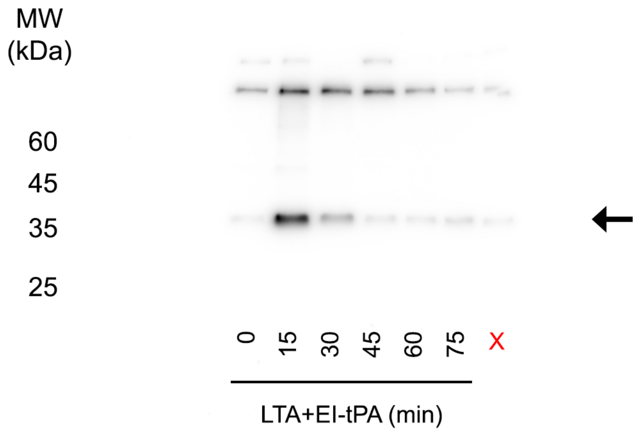

IκBα

39 kDa, 1:1000

Cell Signaling Technology, #9242

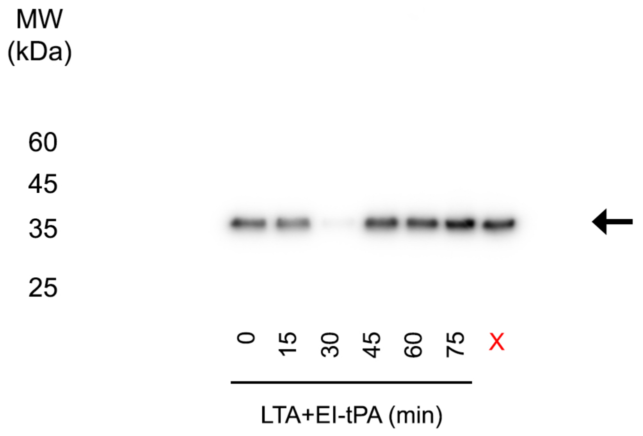

ß-Actin

45 kDa, 1:5000

Cell Signaling Technology, #3700

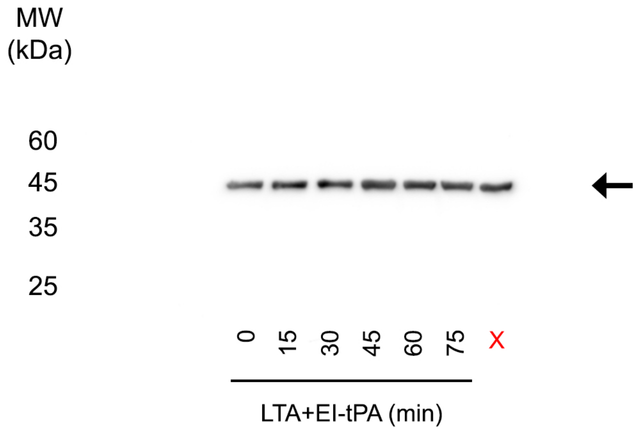

Figure 2, panel E

Digital acquisition: Azure c300 Imaging System

phopsho-IκBα (Ser32)

40 kDa, 1:1000

Cell Signaling Technology, #2859

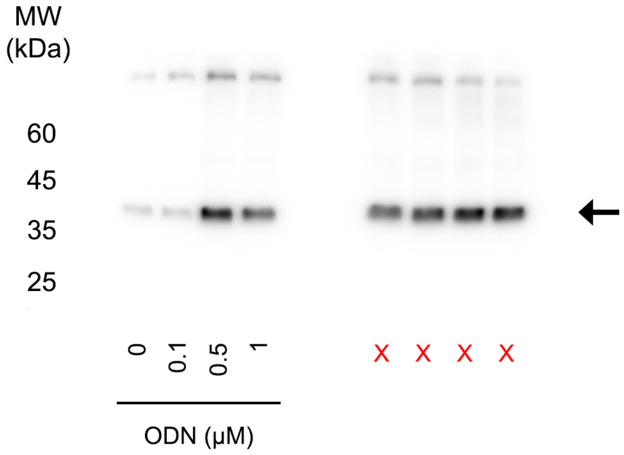

IκBα

39 kDa, 1:1000

Cell Signaling Technology, #9242

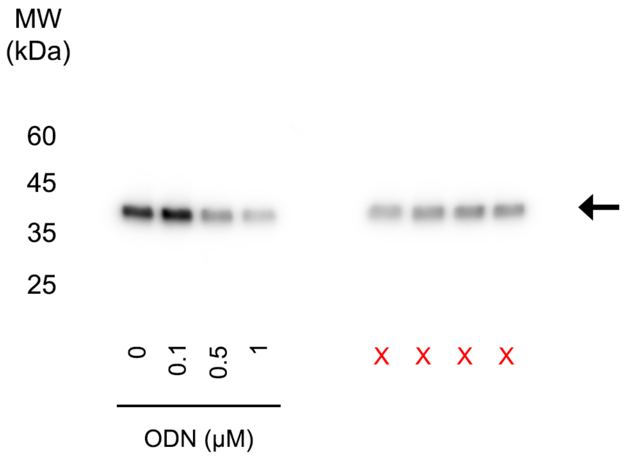

ß-Actin

45 kDa, 1:5000

Cell Signaling Technology, #3700

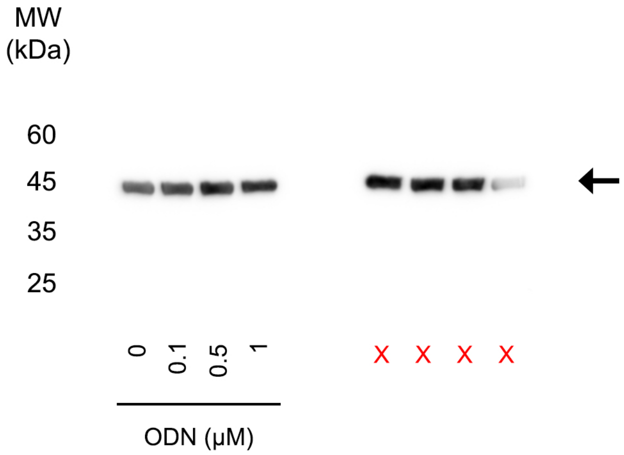

Figure 2, panel F

Digital acquisition: Azure c300 Imaging System

phopsho-IκBα (Ser32)

40 kDa, 1:1000

Cell Signaling Technology, #2859

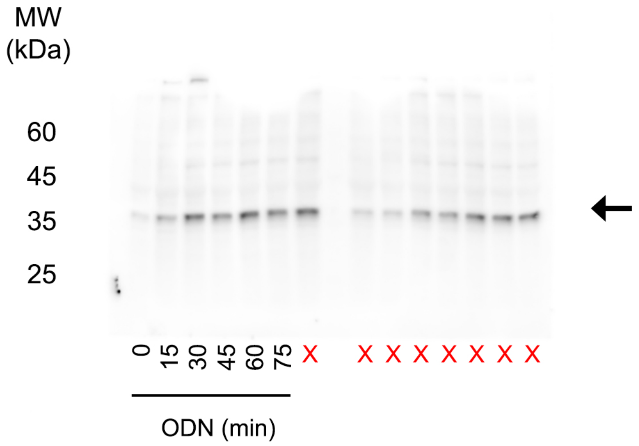

IκBα

39 kDa, 1:1000

Cell Signaling Technology, #9242

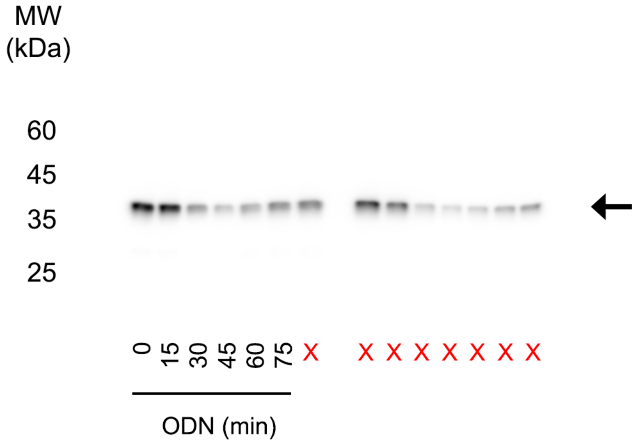

ß-Actin

45 kDa, 1:5000

Cell Signaling Technology, #3700

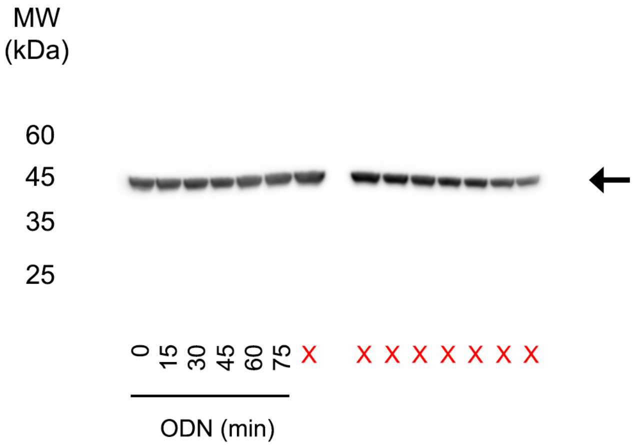

Figure 2, panel G

Digital acquisition: Azure c300 Imaging System

phopsho-IκBα (Ser32)

40 kDa, 1:1000

Cell Signaling Technology, #2859

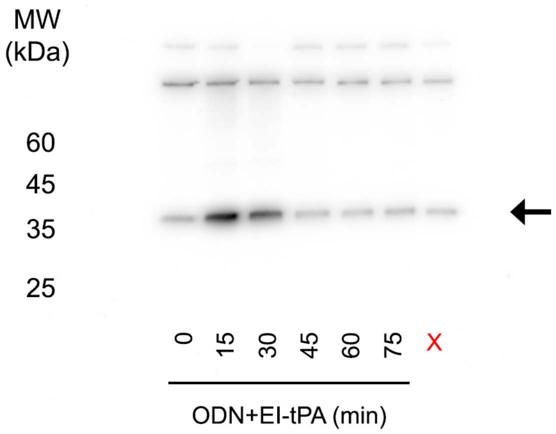

IκBα

39 kDa, 1:1000

Cell Signaling Technology, #9242

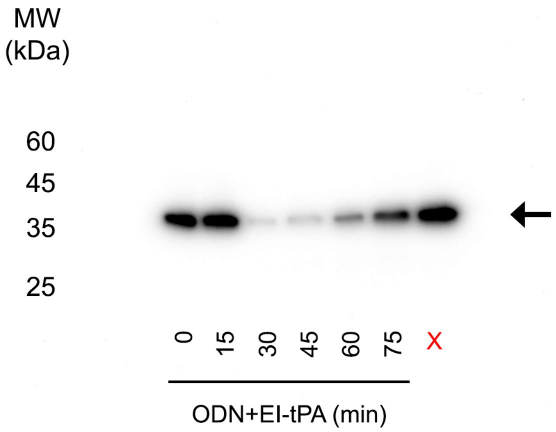

ß-Actin

45 kDa, 1:5000

Cell Signaling Technology, #3700

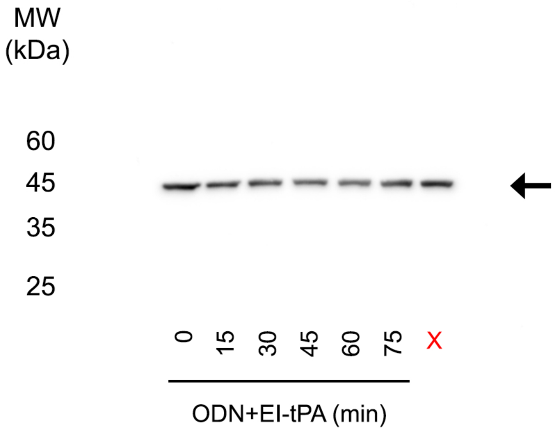

Figure 3, panel C

Digital acquisition: Azure c300 Imaging System

phopsho-IκBα (Ser32)

40 kDa, 1:1000

Cell Signaling Technology, #2859

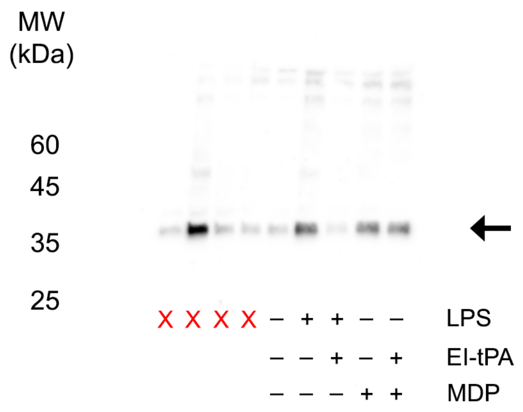

IκBα

39 kDa, 1:1000

Cell Signaling Technology, #9242

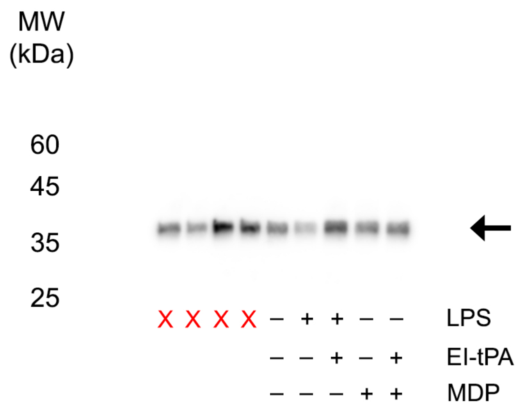

ß-Actin

45 kDa, 1:5000

Cell Signaling Technology, #3700

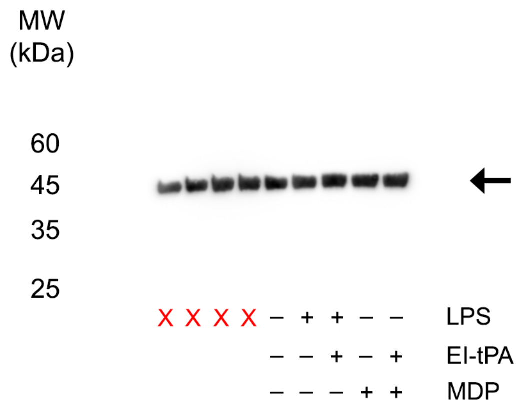

Supplement: S1 Raw images — (PDF) [file pone.0224738.s001.pdf]
